# Mental health information-seeking behaviors and e-health literacy in Tunisian community adults

**DOI:** 10.1192/j.eurpsy.2023.1818

**Published:** 2023-07-19

**Authors:** L. S. Chaibi, F. Fekih-Romdhane, C. Ben Said Saffar, W. Cherif, M. Cheour

**Affiliations:** 1 Psychiatry E; 2 Forensic Psychiatry, Razi Hospital, Mannouba, Tunisia

## Abstract

**Introduction:**

Due to the convenient and easy access to the Internet, there is an increasing tendency to seek online health information instead of formal help-seeking. To date, there is a very little amount of research on online help-seeking behaviors for mental health problems, with no studies having been performed in Tunisia.

**Objectives:**

We aimed to explore mental health information-seeking behaviors and e-health literacy in a sample of Tunisian community adults.

**Methods:**

We performed a cross-sectional descriptive study among adults from the general population. All participants were administered the Barriers to Access to Care Evaluation scale (BACE-30), the Columbia Suicide Severity Rating Scale (SIS-5), the Depression Anxiety Stress Scales (DASS-21), and the eHealth Competency Scale (eHEALS).

**Results:**

A total of 44.2% participants reported having searched the Internet for mental health-related information during the last 12 months. Google was the most used tool by participants when searching for mental health related information. The main topics searched were symptoms and management (n=49%), followed by finding a diagnosis (n=47%), researching for medications and physicians (n=41%). We conducted a multivariate analysis to identify factors related to participants’ e-health literacy. A higher depression score (p=0.037), lower levels of education (p=0.011), and perceived barriers to access to care (p=0.004) were substantially linked to worse e-health literacy.

**Conclusions:**

While a high proportion of participants reported mental health information-seeking behaviors, those with higher depression and who perceived more barriers to care access exhibited lower e-health literacy. These findings may have practical clinical implications.

**Disclosure of Interest:**

None Declared

